# Severe Burn Colonization and Infection: A Single Center Prospective Surveillance Study

**DOI:** 10.1017/ash.2025.419

**Published:** 2025-09-24

**Authors:** Shehryar Malik, Jenny Ziembicki, Christopher Musgrove, Abigail Bornstein, Francesco Egr, Katie Palladino, Nicole Pieri, Meghan Hansen, Selahattin Emin Samasas, Heather Dixon, Mohamed Yassin

**Affiliations:** 1 University of Pittsburgh Medical Center; 2 UPMC Mercy/University of Iowa; 3 UPMC Mercy; 4 UPMC; 5 UPMC; 6 University of Pittsburgh

## Abstract

**Background:** Intact skin is a major barrier for colonization and infections. Burn-related Infections particularly central line associated bacteremia and pneumonia are the most frequent cause of death in burn patients. We aimed at prospectively studying colonization and infections in inpatient burn victims. **Methods:** This is a single center study between April 1st, 2022 – March 30th, 2023. The patients included in the study are patients with inhalational lung injury (IHI) and or patients with total burn surface area (TBSA) of ≥ 10%. All the patients had nasal MRSA swab as well as inguinal swab for MRSA, VRE, C diff, ESBL and CRO screening. These plates were incubated for 2 days at 36 °C. Additionally, fungal cultures were placed on selective fungal media and incubated for 7 days at 32°C. Electronic health records were reviewed for antibiotics used (local or systemic), hospital associated infections (HAI) and clinical and surveillance cultures. The patients were followed for their length of stay (LOS) in the hospital. **Results:** During the study periods, 99 patient encounters were included. There was male predominance (75%) with an average age of 60 years. The average TBSA was 19% and IHI was diagnosed in 41% of the patients with an average length of stay was 28 days. No patients had history of MDRO. MRSA was the most common MDRO identified in clinical and surveillance (routine and study cultures). Groin MRSA was slightly more likely to be positive than nasal MRSA (32% vs. 24% respectively). C diff was positive in 5 cultures and fungal cultures were positive in 4 patients (3 candida and one Aspergillus). Other MRDO (including CRO, ESBL and VRE) were rare (only 2 VRE cultures and no CRO). Local antibiotics were used in the first 10 days in 25% of cases and 35% of the cases after 10 days. Systemic antibiotics were used in 50% of the patients. HAI was diagnosed in 35% patients with pneumonia then urinary tract infections as the most common ones. Blood stream infection was reported in only 2 patients. Burn registry definition of infection included less than half of the patients reported by the National Health Safety Network (NHSN). **Conclusion:** MRSA remain the most important pathogen in burn victims. Strict infection prevention measures and antibiotics stewardship are likely the reason for low infection rate and antibiotic use. NHSN definition may be more sensitive than Burn registry in including HAI in burn victims.

